# Barriers to HPV vaccination in marginalized Roma communities in Slovakia

**DOI:** 10.3389/fpubh.2023.1239963

**Published:** 2023-12-05

**Authors:** Daniela Filakovska Bobakova, Jana Plavnicka, Ingrid Urbancikova, Michael Edelstein, Danielle Jansen, Zuzana Dankulincova Veselska

**Affiliations:** ^1^Department of Health Psychology and Research Methodology, Faculty of Medicine, PJ Safarik University, Kosice, Slovakia; ^2^Olomouc University Social Health Institute, Palacky University in Olomouc, Olomouc, Czechia; ^3^Department of Epidemiology, Faculty of Medicine, PJ Safarik University, Kosice, Slovakia; ^4^Azrieli Faculty of Medicine, Bar Ilan University, Safed, Israel; ^5^Department of Primary and Long-term Care, University Medical Center Groningen, University of Groningen, Groningen, Netherlands

**Keywords:** marginalized Roma communities, ethnic minority, HPV vaccination, health system barriers, access to health care

## Abstract

**Introduction:**

Limited access to healthcare services leads to lower vaccination rates in marginalized Roma communities (MRCs). This study aimed to explore health system barriers to HPV vaccination faced by people from MRCs from multiple perspectives.

**Methods:**

The qualitative study was conducted in Slovakia in 2021/22 with 43 community members and health professionals. Data were analyzed using a combination of content analysis and consensual qualitative research.

**Results:**

A substantial barrier to vaccination is limited coverage of vaccination expenses for certain age categories by health insurance. Moreover, Slovakia faces a significant shortage of healthcare personnel, leading to work overload and a lack of capacity and motivation to address HPV vaccination. Impaired relationships between health care providers and people from MRCs lead to the avoidance of healthcare services, which contributes to insufficient delivery of information and a lack of awareness regarding HPV-related diseases and vaccination.

**Conclusion:**

Strengthening the capacities of health care providers, expanding the age group covered by health insurance and providing tailored information to people from MRCs are necessary prerequisites to increase the availability of HPV vaccination and enable people to make informed decisions about HPV vaccination.

## 1 Introduction

Human papillomavirus (HPV) is one of the most potent viruses associated with multiple cancer incidences (1) and genital warts (2). Although HPV vaccination has the potential to prevent HPV-associated diseases which pose a significant risk to health and constitute a healthcare burden (1, 3), HPV vaccination programs largely differ across the countries and achieving the desired vaccination rates remains a challenge in many of them (4), including Slovakia (5). In Slovakia, HPV vaccination with the bivalent vaccine has been fully covered by health insurance for 12-year-old girls and boys since January 2019 and with the nonavalent vaccine since May 2022 (5). For older age groups, health insurance companies offer benefit programs for optional vaccinations for their clients in which the costs can, under certain conditions, be partially reimbursed (for those 13–18 years old); otherwise, costs for the vaccine have to be fully covered by the recipients (those 18+). In Slovakia, the vaccination coverage rate for 12 years olds in 2021 was 25% for girls and 8% for boys which is less than half compared to neighboring countries such as Hungary or the Czech Republic and far from the desired 90% vaccination coverage rate which was successfully achieved for example in Denmark or Norway (5).

Roma, particularly those living in marginalized Roma communities (MRCs), are among the underserved groups facing limited access to health care and vaccination services, which leads to lower vaccination rates compared to the general population (6). Although there are no data on HPV vaccination rates among Roma, given the low HPV vaccination uptake in the general Slovak population (5) and the lower uptake of other vaccinations in the Roma (6–8), it can be assumed that HPV vaccination rates among Roma living in MRCs are very low. Regions with the lowest HPV vaccination coverage (5) are those with the highest share of Roma population (9).

Lower vaccination rates among Roma can be attributed to discrimination, socioeconomic deprivation, limited access to health care, language barrier, low literacy and low awareness of vaccination as a preventive measure (8, 10). Concerning HPV vaccination specifically, cultural beliefs (no sex before marriage), safety concerns (10), lack of knowledge and poor attitudes and perceptions of HPV vaccination (11), patient-provider relationships and active community health workers, such as community nurses or Roma health mediators (12, 13), might also affect decision-making and uptake in Roma. However, evidence on barriers to HPV vaccination in Roma is scarce and focuses rather on the barriers on the side of Roma than on health system barriers. In a broader sense, however, access to health care is not solely a result of a person’s ability to identify healthcare needs or to seek, reach and obtain healthcare services which is highly influenced by the abovementioned factors. Access to health care results from the interface between these characteristics and the characteristics of health systems, organizations and providers such as approachability, acceptability, availability and accommodation, affordability and appropriateness as described by the conceptual framework of access to health care (14). In European countries with a significant Roma population, including Slovakia, evidence describing health system barriers to HPV vaccination that would inform public health policies and help tackle the vaccination gap is fully lacking.

Therefore, this study aimed to explore health system barriers to HPV vaccination faced by Roma living in MRCs from multiple perspectives. Our objectives were to include perspectives of both healthcare providers and community members and to collect in-depth information about health system determinants of low vaccine uptake (barriers/obstacles) among underserved marginalized Roma communities that could inform the development of new system-level intervention tailored to underserved communities and targeting individual or multiple WHO health system building blocks.

## 2 Materials and methods

### 2.1 Design

A qualitative study exploring health system barriers to HPV vaccination faced by Roma living in MRCs in Slovakia was conducted as a part of the RIVER-EU project (Reducing Inequalities in Vaccine Uptake in the European Region—Engaging Underserved Communities).

### 2.2 Study settings

The study was conducted between October 2021 and May 2022. To capture a wide spectrum of perspectives, participants of multiple expertise were recruited. Purposive sampling was used to involve community members (parents and daughters from MRCs) and health professionals from different backgrounds with a deep understanding of the topic of interest.

A qualitative study with community members was conducted in the Košice district, which has the highest share of marginalized Roma population in Slovakia (9) characterized by high unemployment rates and low educational levels. Three different towns/villages were selected to capture the variation in the share of the Roma population (50–100%), the number (2–10) and size of communities (2,000–7,000 inhabitants), the level of urbanization and separation from the majority population (9).

### 2.3 Sample and procedure

The recruitment of participants was organized in cooperation with Roma health mediators from the national Healthy Communities project using purposive sampling techniques. Roma health mediators are of Roma origin; they live and work in the target communities, know the social structures and local families and have the trust of community members which enabled us to enter the communities and recruit a variety of participants who met the selection criteria. Roma health mediators identified families with girls between 12 and 15 years old and invited eligible parents (both mothers and fathers with different educational levels and employment status) and daughters to participate in the interview with researchers. Semi-structured interviews with participants from the community sample were conducted face-to-face at the community center, or in respondents’ homes.

Purposive sampling techniques were used to recruit professionals involved with the topic of HPV vaccination and working with marginalized Roma communities from Slovakia, preferably from the Košice district, across these categories: general practitioners for children and adolescents, gynecologists, Roma health mediators, public health authorities and policymakers. Participants from the healthcare system could choose the form (face to face, online by Zoom or Teams) of the interviews and where they took place (their office, our office).

Written informed consent was obtained before each interview after a detailed explanation of the project aims and how the data will be treated. In the case of children participating, their parents signed the consent form on behalf of the children. Children were allowed to refuse participation regardless of parental consent. Participants were informed in advance that they are not obliged to answer all the questions and that they can withdraw their consent at any stage.

Participants who agreed to participate were interviewed individually or in groups. The interviews covered topics such as general access to health services, knowledge and attitudes toward vaccination, sources of information, experiences with vaccination, perceived barriers to vaccination and suggestions for improving vaccination services.

### 2.4 Analyses

The transcripts of the interviews processed by a trained research assistant were checked for accuracy and compared with the audio recordings by one of the researchers, who also anonymized the data. We approached the acquired data using a consensual qualitative research (CQR) methodology (15), which requires researchers to reach an agreement on identified topics and interpretations to avoid subjectivity, as different team members performing the analysis provided various perspectives. Data were analyzed on an ongoing basis using a combination of conventional and directed content analysis (16). During data collection, we reached a point of saturation, where no new themes occurred.

The analyses of qualitative data were performed by a team of three researchers with different backgrounds (health psychology, public health, snd social work) and different levels of experience in conducting research with marginalized Roma and with content analyses. Prior to analyses, researchers shared their professional background and personal perspectives and assumptions that might influence their approach to data to acknowledge their subjectivity. Each team member read the transcripts of the interviews and created codes for parts of the interviews independently. Then, the team members met and shared their codes and interpretations to achieve consensus. In the case of differing opinions, the discussion continued until a consensus was reached. We used the MAXQDA software for the coding and analysis process. Based on the codes produced in this data handling, we conducted a content analysis (16). We did this by clustering codes regarding the reported barriers to health care and HPV vaccination. We first read all the codes and sorted them into groups (subthemes) based on the topic they were covering. Afterwards, we created themes by searching for an umbrella concept for the subthemes.

As a last step, the identified themes covering different health system barriers were then sorted depending on how they corresponded with six core components or “building blocks” of the WHO health systems framework (17): (1) service delivery, (2) health workforce, (3) information, (4) medical products, (5) vaccines and technologies, (6) financing and leadership/governance. This framework was identified as being suitable for organizing the identified health system barriers, as it was designed to address monitoring and evaluation needs for different users and multiple purposes (17).

## 3 Results

The sample consisted of three groups of respondents with different perceptions of the studied topic. We interviewed 13 teenage girls, 17 parents (mostly mothers) and 13 professionals with various expertise at different levels of the work hierarchy. Most of the respondents (28) were interviewed individually. Fifteen respondents were interviewed in small groups or couples. The girls and their parents were all residents of MRCs. Also, six professionals were of Roma origin. The background characteristics of the sample can be found in Table 1.

**Table 1:** Background characteristics of the sample.

	Daughters (*N* = 13)	Parents (*N* = 17)	Professionals (*N* = 13)	Total (*N* = 43)
Gender	N (%)	N (%)	N (%)	N (%)
Male	0 (0)	2 (11.8)	5 (38.5)	7 (16.3)
Female	13 (100)	15 (88.2)	8 (61.5)	36 (83.7)
Age	Mean (range)	Mean (range)	Mean (range)	Mean (range)
	13.9 (12–15)	41.4 (33–54)	46.9 (30–68)	34.7 (12–68)

The following sections describe the main themes regarding health system barriers to HPV vaccination according to WHO building blocks. An overview of the identified barriers per health system building block can be found in Table 2.

**Table 2:** An overview of identified barriers per health system building block.

Health system building block	Identified barriers
Leadership and governance	(a) Insufficient coordination between stakeholders engaged in vaccination
	(b) No awareness-raising of HPV
	(c) Lack of government intervention to address the influence of anti-vaccination movements
	(d) Unresponsiveness and passivity of the system
Health workforce	(a) Healthcare professionals are insufficiently trained and skilled to provide tailored information about vaccination
	(b) Discrimination against target population
Service delivery	(a) Insufficient access to services
	(b) Insufficient resources to deliver all vaccines
	(c) Lack of, insufficient or inadequate delivery of information
	(d) Language barriers
Financing	(a) Costs to the systems
	(b) Costs to the patients
Medical products	(a) Optional status of HPV vaccine
Health information systems	NA

### 3.1 Leadership and governance

#### 3.1.1 Insufficient coordination between stakeholders engaged in vaccination

A lack of coordination between different organizations delivering vaccines and/or a lack of coordination between different elements of the healthcare system pose a significant barrier according to the respondents. Roma health mediators are perceived as an essential bridge between the community and health services, yet coordination between the mediators and healthcare workers is not optimal.

*“I think that they* [doctors] *need really, very, very good cooperation with those Roma health mediators.* […] *Those Roma health mediators, I know it’s difficult, but in my opinion, they should be educated at a slightly higher level about some activities. So, I would call it something between, um, between the nurse and the social worker directly in the community, who then cooperates with that doctor. Right? So, if this were the case, then the cooperation and also the health care in those communities would be, um, much better in my opinion.*”

(primary paediatrician)

#### 3.1.2 No awareness raising of HPV

The healthcare system is not able to reach MRCs with relevant and sufficiently formulated information about HPV infection, HPV-related cancer and HPV vaccination. People from MRCs are not reached by media campaigns. Also, schools, municipalities or health insurance companies are not involved in providing tailored information to relevant age groups, and the topic of HPV is not on the agenda of Roma health mediators.

“*Insurance companies should promote this vaccination; it should be talked about everywhere, on the radio and TV, maybe even in those children’s programmes.* […] *It’s definitely not talked about much, it’s not talked about, it’s not written about, and it’s not in the media, so push it in that direction. And mainly push it to those people that it’s already paid for, that it’s free.*”

(gynaecologist)

“*Well, HPV is, let’s say in general, very little talked about, very little communicated in the Roma population in the localities.* […] *I would welcome more such education and training. First of all, I would like our workers to know more about it, so that they could inform, talk to and advise people in their localities about what and how so that they would not underestimate it.*”

(coordinator of Roma health mediators)

#### 3.1.3 Lack of government intervention to address the influence of anti-vaccination movements

The healthcare system is not able to fill the space with relevant information, and more space is given to anti-vaccination movements and the dissemination of false and contradictory information on the Internet, social media and among the lay public. Knowledge and attitudes of people from MRCs are largely influenced by information that reaches them from various sources.

“*I perceive from my experience that there is a certain gap in the healthcare ‘market’, which is then filled by all kinds of charlatans and spreaders of various delusions, I'll put it bluntly.* […] *it’s not systematically covered or covered enough, and to be fair, either it creates that gap that isn’t filled with anything – that information doesn't come – or it will be filled willingly and gladly simply by those who benefit from it in some way.* […] *They are very successful and therefore one of the effects is absolute distrust in science as such and in scientists, which is a disaster.*”

(public health authority)

#### 3.1.4 Unresponsiveness and passivity of the system

Respondents suggested that underserved populations require proactive, tailored approaches for vaccination. In Slovakia, health care providers are mostly convinced that the healthcare system is built and functional, and the only thing people need to do is to come and engage with it. A lack of engagement from people living in MRCs is perceived as a barrier to access rather on their side than on the side of the healthcare system.

“*I think that the barrier is mutual, that it is literally some kind of ‘ditch’, where on the part of the system there is such a deep conviction that it is enough if the system is technically built. That is, there will be some network of buildings, which is filled with outpatient departments; there are doctors in those departments; those doctors have opening hours; those opening hours are best posted on the door and you just have to come there and things will happen.* […] *Probably the most common thing I heard was: ‘We are here and they have no other task but to come and participate in those processes, and they are not willing to do this either’.* […] *And all the rest is already on the side of the recipient of that ‘service’ because, from the provider’s side, it is a done deal.*”

(public health authority)

### 3.2 Health workforce

#### 3.2.1 Healthcare professionals are insufficiently trained and skilled to provide tailored information about vaccination

Insufficient training and skills of health care providers as a barrier concern their knowledge about HPV and HPV vaccination, their cultural competency and their assumptions about the target population. Vaccine hesitancy on the part of health care providers or their non-participation/inactivity in HPV vaccination is, according to some of our respondents, influenced by the religious beliefs of health care providers, their preference for other means of prevention (sexual restraint), the influence of hoaxes, insufficient information about HPV vaccination among health care providers and their lack of motivation to discuss and offer HPV vaccination to patients.

“*I don't know what percentage are of retirement age. So, they are happy to be able to handle the basics and maybe because they are older, maybe because of the HPV vaccine, which is a relatively new thing and modern, so maybe they don’t believe it. So, they do not devote themselves to it and do not act actively in this area to somehow actively persuade those people.* […] *Maybe they don’t have enough knowledge about the vaccination, what it actually is and why and how and that the insurance company already covers it.* […] *But some are anti-vaxxers against it.*”

(gynaecologist)

“*And then we have those in the population who say there is another way of protection; let’s call it sexual restraint, and regular preventive examinations, so many paediatricians have the same opinion, huh?* […] *That they are, let’s say, I will call it more religious because I encounter it the most, so they are so reserved that they may not actively offer it* [the HPV vaccine].”

(primary paediatrician)

Health care providers are not providing detailed information humanly but are talking rather technically, which might have an impact on decision-making.

“*They* [Roma] *have distrust; they are afraid.* [*…*] *I think that* [because of] *the approach of doctors, because they are not given the exact information, hey, what is it actually about, that it can’t cause anything bad, only good.* [*…*] *Because you need to pay a little more attention to the Roma so they understand what it is all about.* [*…*] *Because most Roma won’t understand technical words, right? So they don’t know what it’s about and say ‘No, we don’t want to.’ Right? Maybe she would go for it or he would go for it, but it wasn’t explained in detail, it wasn’t said humanly, hey, but professionally.*”

(Roma health mediator)

#### 3.2.2 Discrimination against the target population

An impaired relationship and insufficient or inappropriate way of communication between health care providers and patients from MRCs is burdened by prejudices, impersonal attitudes and double-standards. This was indicated by respondents from each group. According to health professionals, this creates a barrier for marginalized Roma in access to health care and has an impact on the perceived quality of health care. Many professionals, but also mothers from MRCs, indicated that it might lead to avoidance of health care.

“*People from the socially weaker strata have very bad experiences with doctors, because those doctors are sometimes literally arrogant; they are not at all interested in the person who has a problem; they also have bad experiences with nurses who beat them back very quickly, forcefully and literally brutally.*”

(Roma health mediator)

“*Um, what would help me? What would help me? So, that they respect us and our children, and they should also consider our children. No, no offence, ma’am, but you shouldn’t judge what a white child is like and this is a Romani child. They should deal with and take children the same way whether he or she is white or black; that’s all I'd like, that they have respect for our children as well.*”

(mother from an MRC)

“*She* [the doctor] *is rude to some people, but sometimes she is also rude to her nurse. Her mood changes.*”

(girl from MRC)

Both mothers and daughters from MRCs described situations of interactions with their doctors as unpleasant. As a reason for that, they often provided their opinion that doctors have negative attitudes toward them because of their origin. Racism was explicitly mentioned several times.

“*Well, the doctor is good, but the nurse, I don’t even know how to tell you.* [*…*] *She doesn’t know … she doesn’t know how to deal with people.* [*…*] *Maybe she’s racist or I don’t know what she is.*”

(mother from an MRC)

*“Because I'm a Roma woman, because we’re Roma, but because they put us aside, sit down away from whites. I also had personal experience with it, and they knew that I was employed; they saw that I was clean, that I was decent, I have a card* [health insurance]*, I can express myself, and I also had such a problem. That I say from my own experience.”*

(Roma health Mediator)

### 3.3 Service delivery

#### 3.3.1 Insufficient access to services

According to our participants, access to healthcare services is limited due to various reasons, including physical access and the complexity of navigating the system and booking appointments. Primary care outpatient clinics are often several kilometers from MRCs and with bad traffic connections.

“*So, well, if I were to take the bus, it would be at eight o’clock and I would wait there until half past twelve before going home.*”

(mother from an MRC)

“Where should she put the seven children when she needs to go 40 km or more to the nearest workplace? What will she do with them and where will she get the money? And another thing, she has to get off somewhere at a station in a city she has never been to in her life, she has to find her way there, she has to know how to get on the right train or bus, and she has no idea how to do it, she doesn’t know where to buy tickets. These are things that are completely legitimate.”

(public health authority)

The process of HPV vaccination generates major logistical barriers, as it requires parental consent, the presence of a legal guardian during vaccination, a prescription from a pediatrician, ordering the vaccine from a supplier and the patient picking up the vaccine him/herself at the pharmacy before bringing it back to the health center. The whole process is repeated for the second dose. This process is even more complicated in rural areas where pharmacies are not present in each village.

“*The vaccination procedure* […] *can’t be done that way. Not only that you have the prescription and you need to go 20 km to the pharmacy with the prescription and they say they don’t have it.*”

(public health authority)

#### 3.3.2 Insufficient resources to deliver all vaccines

Health care providers highlighted their restricted capacity and the high number of patients per pediatrician, leading to a lack of pediatricians’ time, energy and motivation to address unnecessary or non-compulsory tasks, such as HPV vaccination and prioritizing acute management of ill children, mandatory preventive check-ups and vaccinations which take up all their capacities.

“*Before the pandemic* […] *I myself had the energy to talk to people about optional vaccinations at my clinic, but now I’m changing my mind and saying that it’s not possible, right? By the paediatrician. I don’t have the time, space, energy, or motivation to argue with them, do I? I just don’t have the drive for it anymore, right? And that’s why, with the workload that the paediatricians have* […] *I’d rather examine 5 more sick children because I don’t have time for those either.*”

(primary paediatrician)

“*You know what? I don’t really offer it* [HPV vaccine]*, because I have so many children that I have a problem vaccinating them with mandatory vaccinations.*”

(primary paediatrician)

This lack of capacity was also reported by community members.

“*We don’t have a normal doctor, that we go to the doctor’s office, that we sit down, that we talk, like about children. When we go there, everything is fast, because we don’t have a doctor in the village* […]. *She doesn’t have the time to communicate with us.* [*…*] *She keeps saying that she has many patients.*”

(mother from an MRC)

Moreover, a large proportion of doctors are of retirement age, which might cause the situation to be even worse in the upcoming years. The insufficient capacity was compounded by the difficulty in attracting new health care providers to the catchment areas with MRCs, because of insufficient support, financial motivation and salary conditions.

“*I see the care of MRCs as a huge problem also from the perspective that there is a huge shortage of primary paediatricians. Fifty percent of primary paediatricians are of retirement age, and the young ones will simply not go to those villages and those MRCs voluntarily to become doctors.* […] *Given the conditions that the doctors have there, I mean the overall conditions, hey? Not only financially, but also mentally, including the number of children, payment mechanisms, and the difficulty of the work and communication with different types of people, so they are undervalued and, in my opinion, they are doing their best.*”

(primary paediatrician)

#### 3.3.3 Lack of, insufficient or inadequate delivery of information

The previously mentioned restricted capacities of health care providers are leading to a situation in which people from MRCs are not informed about HPV infections by their doctors and are not offered HPV vaccination. The information does not reach them from any other source either, and as a result, they are unaware of HPV infections and its association with cancer, or of how vaccination (not only HPV but generally) works and whether it is effective.

*“Doctors don’t even talk about it* [HPV]*; they don’t talk about it in the news or outside, that’s why* [people do not know about it].”

(girl from MRC)

“*He* [the doctor] *is the main one, yeah, who is supposed to provide information or tell them that ‘this will help you’, ‘that will help you’; he should do all that, but it’s not like that, because the district physician will say: ‘I still have 20 patients outside, yeah, I won’t deal with you.’.*”

(Roma health mediator)

At the same time, both parents and girls often indicated that their doctor is the most reliable source of information for them and if they would like to get more information about cervical cancer or HPV vaccination, they would most likely approach their doctor.

“*I would go to the doctor to find out what it is and how it can be treated and so on. Only them, because they* [doctors] *know more than Google, Facebook, or friends.*”

(girl from MRC)

According to our participants from the group of professionals, the existing field services providing health mediation in MRCs have insufficient personal capacities to be able to cover the topic of HPV prevention on top of more pressing issues related to the health of people living in MRCs. Also, the coverage of these services is limited, as they are not available to all people in need of such services.

“So, I think there is a need to increase the number of Roma health mediators in the given communities. So, one paediatrician usually cooperates with one such Roma health mediator; they should actually have one in every marginalized community.”

(primary paediatrician)

*“Rather, we are focusing on ensuring that children are vaccinated* [with mandatory vaccines]*, and we are trying to ensure that the gynaecological check-ups are carried out, that women go to them regularly and that they also have a preventive check-up at the district doctor and so on, hey? That’s enough; we have enough to do.”*

(Roma health mediator)

#### 3.3.4 Language barriers

Language as a barrier includes difficulties in communication during consultations as well as the unavailability of information in the preferred language. Respondents mentioned that leaflets and health care providers use language and expressions which are hard to understand, and information materials do not align sufficiently with the needs of the people from MRCs. Information is tailored to middle/higher class educated women.

“*Well, they also write something like that, as you say, uterus, to vaccinate, or whatever the substance is there. Well, sometimes I don’t understand at all what it is about.* […] *Sometimes they also write in Latin, so we won’t understand what is there at all.*”

(mother from MRC)

“*Well, the difference is that if a non-Roma comes to talk to them* [Roma] *about it* [HPV]*, in most cases 50% of those people will not know what they were talking about, or they will not understand what they were talking about. And the difference is that they have a little more trust in me, and I can say it in both the Romani language and the Slovak language.*”

(Roma health mediator)

Several respondents indicated that people with lower literacy and limited capacity for understanding information related to health might confuse the abbreviation “HPV” with “HIV.” The translation may not be the right solution, as for many expressions there are no alternatives in the Romani language, as the vocabulary is limited.

“*Once I read about vaccination against jaundice, then that there is a vaccination against HIV positive, that is the AIDS I mentioned before. Yeah. I read about several, yeah, but … as one says to himself, hey, it doesn’t concern me yet, so…*.”

(mother from MRC)

### 3.4 Medical products

#### 3.4.1 Optional status of the HPV vaccine

Mothers from MRCs differentiated between routine vaccines, such as MMR, and “optional” ones, such as HPV. “Optional” vaccines were viewed by them as inferior and somehow suspicious. Once the vaccination is optional, many people do not even consider it.

“*I am not at all interested in optional vaccinations, nor would I have children vaccinated at all. If it is not mandatory, I will not vaccinate them. I wouldn’t even vaccinate myself.* [*…*] *I don’t know, maybe it’s not even explained well.*”

(mother from MRC)

Healthcare professionals reported that people from Roma communities are not able to understand the meaning of vaccination as a means of prevention, and the optional status of HPV vaccination is a barrier.

*“It’s as if they can’t fully evaluate the benefit in advance.* […] *when you inject their child, who according to them is completely healthy, and you say that you are doing it so that something will not happen to him in 10 or 15 years, it is as if they have a problem evaluating why. So, the barrier is huge there. So, in my opinion, the only thing that would help there would be if the vaccination were mandatory, nothing else would help there.*”

(primary paediatrician)

### 3.5 Financing

#### 3.5.1 Costs to the system

Another substantial health system barrier to vaccination mentioned by respondents is the high cost of the HPV vaccine and the limited coverage of vaccination expenses from health insurance. This is likely to be caused by the high price of the vaccine and the willingness of health insurance companies to reimburse high costs.

*“They* [health insurance companies] *are aware of the need (HPV vaccination) and what it is against, but then when the economic side comes into play, they look at it differently.* […] *On the one hand, they know that it is necessary, on the other hand, it really costs a lot of money, because it is really one of the most expensive vaccines, right?”*

(primary paediatrician)

#### 3.5.2 Costs to patients

HPV vaccination is fully covered by health insurance companies only for 12-year-old girls and boys. This interval is perceived by respondents to be very short for people from MRCs to be able to organize vaccination fully covered by health insurance for their children in case of interest. Out-of-pocket payment to obtain vaccination outside of the regular reimbursed scheme and to cover travel costs pose an additional financial barrier for people from MRCs.

*“Since it is paid out of pocket* [in adulthood] *and not covered by public insurance somehow, these marginalized groups are not interested in such vaccinations because it is very expensive.”*

(gynaecologist)

“*For example, there are families that do not work. They only have those social benefits in material need and they have several children, so they also have to feed the children and clothes, and everything, and if the woman needs the vaccination and has nothing to pay for it, what is she supposed to do?*”

(mother from MRC)

“*It would be better to give … I don't know how to say it … this reimbursement* [full coverage of the vaccine from health insurance]*, or whatever you call it. This, for all girls to have. Both adults and children.*”

(girl from MRC)

## 4 Discussion

This study aimed to explore health system barriers to HPV vaccination faced by Roma living in MRCs in Slovakia from multiple perspectives. The identified health system barriers seem to be related to the utilization of health care in general, as well as to the HPV vaccination specifically.

### 4.1 Leadership and governance

The current state of the health system and its approach to HPV vaccination requires quite proactive attitudes of health care users to obtain information, make a decision and get vaccinated against HPV. Insufficient regulation of information on the Internet and social media gives space to anti-vaccination movements which encourage mistrust of vaccination as a tool for prevention. Although media campaigns have recently relaxed since the nonavalent vaccine began to be fully covered by health insurance for 12-year-old boys and girls, these target the general public and information provided in the limited space of ads in public and social media is not sufficient to make an informed decision and require a further active search for information. This is not likely to be efficient in settings with limited resources and limited access to information, health care and low health literacy, causing a limited ability to understand and evaluate health-related information, which is a characteristic often associated with Roma, as has been described by health professionals in our research as well as elsewhere (18). Underserved populations require more proactive, tailored approaches for vaccination; however, the responsiveness of the healthcare system seems to be low toward people living in MRCs.

### 4.2 Health workforce and service delivery

Generally, access to health care was considered to be limited; however, perceptions of the causes differed among the groups. People from MRCs are affected by the restricted capacities of primary care providers and perceive the tension it causes. Moreover, they also perceive the healthcare system as unfriendly and interaction with health care providers often as unpleasant due to their attitudes and behaviors influenced by prejudices and racism. Impaired relationships with health care providers contribute to the avoidance of healthcare services, which was similarly described by previous research (19, 20). More equitable access for Roma could be ensured by establishing a respectful and understanding relationship between health care providers and their Roma patients (21).

According to health professionals, the network of primary health care providers, including the number of primary care pediatricians, is insufficient. Many of them are of retirement age, and the number of patients per pediatrician is high. This is consistent with the findings of a spending review (22), according to which primary care is underfunded and less developed at the expense of specialized care. The availability of primary health care in Slovakia is poor and less effective also due to insufficient competencies, capacities and the structure of staffing (22). Restricted capacities cause pressure on the outpatient departments and limit access to health care, which was viewed as a universal problem concerning all patients, not only those from MRCs. However, this problem is more prominent in rural areas (22–24), especially in areas with a higher proportion of MRCs (24). The healthcare system is unable to attract a new healthcare workforce to such areas and offer the conditions and support (not only in terms of finances) needed to overcome difficulties connected to the demanding work with the target population and the administrative burden. Attracted by higher wages abroad, students in medical fields, including nurses, leave Slovakia after graduation to pursue their careers elsewhere (23). This complex situation leads to work overload and a lack of capacity and motivation to address HPV vaccination in primary care pediatricians.

Roma health mediators in MRCs focus on awareness-raising activities, serve as a bridge between health care and MRCs and help to overcome barriers in access to health care (25). Although the number of Roma health mediators and the number of communities in which they are operating is gradually increasing, this support is not available to all communities in need of such services, and the capacities of Roma health mediators are greatly utilized by helping to ensure basic health care, mandatory preventive examinations and mandatory vaccinations of children, acute health conditions and outbreaks (26). According to the Annual Report of Healthy Regions (26), which operates the health mediation program in Slovakia, educational activities mainly focus on child care and women’s health as well as hygiene and prevention of infectious diseases causing outbreaks; thus, HPV is not yet on the agenda of Roma health mediators.

Lack of capacities and motivation of health care providers who are perceived as the most important source of information to address HPV vaccination leads to a lack of awareness about HPV-related cancer and its prevention among community members. This is in line with recent findings on barriers to HPV vaccination experienced by racial/ethnic minority groups indicating that HPV vaccine uptake is associated with a lack of recommendations for HPV vaccination from health care providers, mistrust toward healthcare professionals and low awareness of HPV and HPV vaccination (27, 28). Moreover, the process between the decision to vaccinate and the actual vaccination is complicated for people with lower literacy, given the number of steps that separate them. Problems with navigation in the healthcare system occur in people with lower health literacy in vulnerable and underserved populations (29) such as Roma (18, 20).

### 4.3 Medical products and financing

Another group of barriers results from the way HPV vaccination is organized and financed. As many Roma living in MRCs suffer from financial hardship, those who are not eligible to have HPV vaccination fully covered by health insurance cannot afford it. The price of the vaccine leads to the hesitancy of health insurance companies to widen the age interval for free vaccination from 12-year-old to 9–15-year-old boys and girls despite pressure from the Ministry of Health and recommendations of health care providers (5). Although the vaccine itself is free for 12-year-old boys and girls, many primary pediatricians charged patients a fee for administering the vaccine, which was heavily criticized by health professionals, the public and the media (23). Such additional costs, together with travel costs, pose another barrier in access to HPV vaccination. However, from April 2023, all health insurance companies now reimburse the performance of HPV vaccination of 12-year-old children, which should lead to the cancelation of the fee for the administration of the vaccine by primary pediatricians. This measure has the potential to improve the affordability of HPV vaccination by eliminating out-of-pocket payments. If people from the MRCs were better informed about the possibility of getting vaccinated for free, it could support the acceptance of the HPV vaccine. However, without improving awareness, this measure by itself is unlikely to have much effect in marginalized Roma communities.

### 4.4 Strengths and limitations

The strength of this study can be seen in the diversity of the sample involving a range of stakeholders from community members to health care providers and policymakers with different perspectives on the studied topic, thus ensuring a rich spectrum of viewpoints. Moreover, following the principles of the Consensual Qualitative Research (CQR) methodology (15) was an important step in avoiding the subjectivity of researchers and improving the reliability of the results and interpretations. The use of the WHO health system building blocks framework allowed us to focus on health system barriers and organize them accordingly to highlight the essential components of a health system in which barriers are operating and need to be addressed.

A limitation of this study may be seen in the purposive sample consisting mostly of women. This limitation may be attributable to the fact that the expert field concerning the topic of interest is limited and feminized in Slovakia, and the HPV vaccination is mostly viewed as a prevention of cervical cancer and consequently as concerning more women than men. Moreover, in marginalized Roma communities, it is usually mothers who are responsible for dealing with issues related to health, and thus, they are more willing to participate in discussions concerning health. Another limitation of the study is that it was conducted in one specific region. MRCs in Slovakia are very heterogeneous; thus, the results should be generalized with caution. The selected region is the one with the highest share of the Roma population and at the same time the lowest HPV vaccination rates, which is a good prerequisite for obtaining relevant data about barriers to HPV vaccination in MRCs. However, in other regions with varying demographics and healthcare resources, the barriers to HPV vaccination might occur to a lesser extent.

### 4.5 Implications

Identified health system barriers related to the utilization of health care, in general, are essentially connected with financial and legislative frameworks. It is necessary to increase the number of primary care providers, improve the system of financing and reimbursement of their services, provide them with sufficient support and secure decent working conditions, reduce the administrative burden associated with operating clinics and attract primary health care providers to less attractive regions. Increased capacities and motivation of primary care providers to address HPV vaccination are essential to increase vaccination rates not only in MRCs but in the whole population. Training of healthcare professionals might be needed in the area of communicating with people with lower health literacy, as the provision of tailored and understandable information has an impact on decision-making. The HPV vaccine fully covered by health insurance can be currently prescribed only by primary pediatricians. Relaxation of this restriction, optimally without limitation, might add some capacities of other health care providers, such as gynecologists.

Underserved groups, such as Roma, require special attention and proactive outreach to provide vaccination (6). Increasing the capacities of Roma health mediators, extending their scope to other communities in need of such services and providing them with training on HPV vaccination might partially take the burden off the shoulders of health care providers and help to overcome the language barrier. Roma health mediators can provide Roma living in MRCs with tailored and understandable information and help people navigate the complicated vaccination process. It would also be appropriate to consider how this process can be simplified. Expanding the age group for which the vaccine would be covered by health insurance and distributing the vaccine from the pharmacy to the care provider without involving parents or establishing school vaccination programs would contribute to simplifying the vaccination process and increasing the availability of the vaccine. These changes are of a legislative nature and create pressure on the Ministry of Health and health insurance companies, but pressure should also be exerted on pharmaceutical companies, too, as the price of the HPV vaccine is high and its reduction would mean better accessibility for several socioeconomic and age groups that are not covered by health insurance.

Future research should focus on the development and evaluation of tailored interventions for underserved groups, including communication strategies to foster their positive attitudes toward vaccination care as a means of prevention. Moreover, it is necessary to assess the needs of health care providers to enable them to communicate the topic of HPV and provide high-quality health care to patients with different needs.

## 5 Conclusion

Our results suggest that the healthcare system is not able to provide high-quality services to people with different needs, and their overall access to health care is limited. Slovakia faces a significant shortage of healthcare personnel, leading to work overload and a lack of capacity and motivation to address HPV vaccination. Impaired relationships between health care providers and their patients from MRCs lead to the avoidance of healthcare services. Moreover, the healthcare system fails to reach MRCs with appropriate and understandable information about HPV-associated diseases and HPV vaccination, which contributes to extremely low awareness of HPV, related cancer and possible prevention strategies. Limited coverage of vaccination expenses from health insurance makes HPV vaccination inaccessible for people from a disadvantaged background, which is often the case for Roma living in MRCs. Strengthening the capacities of health care providers, expanding the age group covered by health insurance to at least 9–15 years and providing tailored information to people from MRCs are necessary prerequisites to increase the availability of HPV vaccination and enable people to make informed decisions about HPV vaccination.

## Data availability statement

The raw data supporting the conclusions of this article will be made available by the authors, without undue reservation.

## Ethics statement

The studies involving humans were approved by Ethics Committee of the Faculty of Medicine at PJ Safarik University under number 11 N/2021 and Ethics Committee of Košice Self-governing Region under the protocol identifier “H2020 RIVER-EU.” The studies were conducted in accordance with the local legislation and institutional requirements. Written informed consent for participation in this study was provided by the participants’ legal guardians/next of kin.

## Author contributions

DF, DJ, and ME: conceptualization. DJ and ME: methodology. DF, ZD, JP, and IU: formal analysis, investigation. DF: writing—original draft preparation and writing—review and editing. ZD, DJ, and ME: supervision. All authors have read and agreed to the published version of the manuscript.
